# Supplementary material for: A New Approach for Understanding International Hospital Bed Numbers and Application to Local Area Bed Demand and Capacity Planning
Source: Int J Environ Res Public Health. 2024 Aug 6;21(8):1035. doi: 10.3390/ijerph21081035 (PMC11353596; doi:10.3390/ijerph21081035)
Supplement: Supplementary file 1 [file ijerph-21-01035-s001.zip › ijerph-3044594-supplementary.pdf]

Supplementary Material

Table S1. Available bed numbers, average occupancy, and lines of constant turn-away.

Beds	0.001% Turn-away	0.01% Turn-away	0.1% Turn-away	1% Turn-away	3% Turn-away	5% Turn-away	20% Turn-away	50% Turn-away
1	0.0%	0.0%	0.1%	1.0%	3.0%	5.0%	20.0%	50.0%
2	0.2%	0.7%	2.5%	7.5%	13.6%	17.9%	40.0%	68.3%
3	1.3%	2.9%	6.3%	15.2%	23.1%	28.3%	51.5%	76.5%
4	3.2%	5.9%	11.0%	21.6%	30.5%	35.8%	59.0%	81.3%
5	5.0%	9.0%	15.2%	27.0%	36.4%	41.9%	64.2%	84.4%
6	7.8%	12.1%	19.2%	31.6%	41.0%	46.5%	68.1%	86.7%
7	10.4%	15.0%	22.6%	35.5%	44.9%	50.4%	71.2%	88.6%
8	12.5%	17.8%	25.6%	38.9%	48.3%	53.5%	73.7%	89.4%
9	15.0%	20.3%	28.4%	41.7%	51.1%	56.3%	75.7%	90.6%
10	17.2%	22.6%	30.9%	44.2%	53.6%	59.1%	77.5%	91.4%
11	18.7%	24.7%	33.2%	46.6%	55.7%	60.7%	79.3%	92.3%
12	20.6%	26.8%	35.3%	48.7%	57.6%	62.5%	80.0%	92.5%
13	22.7%	28.5%	37.2%	50.5%	59.3%	64.1%	81.2%	93.1%
14	24.2%	30.3%	38.9%	52.2%	60.8%	65.6%	82.3%	93.6%
15	25.5%	31.9%	40.5%	53.5%	62.4%	67.3%	83.2%	94.0%
16	26.8%	33.4%	42.0%	55.1%	63.5%	68.0%	84.0%	94.4%
17	28.1%	34.8%	43.4%	56.4%	64.9%	69.1%	84.7%	94.7%
18	29.5%	36.1%	44.7%	57.6%	65.6%	70.1%	85.3%	95.0%
19	30.8%	37.3%	45.9%	58.7%	66.7%	71.1%	85.9%	95.3%
20	32.1%	38.5%	47.0%	59.6%	67.9%	72.4%	86.5%	95.4%
21	33.1%	39.6%	48.1%	60.7%	68.7%	72.7%	86.9%	95.7%
22	34.1%	40.7%	49.1%	61.6%	69.5%	73.5%	87.6%	95.7%
23	35.2%	41.7%	50.1%	62.5%	70.3%	74.2%	88.0%	95.9%
24	36.2%	42.5%	51.0%	63.3%	71.0%	74.8%	88.3%	96.0%
25	37.2%	43.5%	51.8%	63.9%	71.7%	75.9%	88.7%	96.3%
26	38.0%	44.2%	52.7%	64.8%	72.2%	76.0%	88.9%	96.3%
27	38.8%	45.2%	53.5%	65.5%	72.8%	76.5%	89.5%	96.5%
28	39.6%	46.1%	54.2%	66.1%	73.3%	77.1%	89.7%	96.6%
29	40.5%	46.9%	54.9%	66.8%	73.7%	77.5%	89.9%	96.7%
30	41.3%	47.5%	55.6%	67.1%	74.6%	78.5%	90.2%	96.9%
31	41.9%	48.1%	56.3%	67.9%	74.9%	78.4%	90.6%	96.9%
32	42.6%	48.8%	56.9%	68.4%	75.3%	78.9%	90.8%	97.0%
33	43.2%	49.4%	57.5%	69.0%	75.7%	79.2%	90.9%	97.1%
34	43.9%	50.0%	58.1%	69.4%	76.3%	79.6%	91.3%	97.2%
35	44.6%	50.7%	58.6%	69.7%	76.8%	80.6%	91.4%	97.3%
36	45.1%	51.4%	59.2%	70.4%	76.9%	80.3%	91.6%	97.4%
37	45.7%	51.9%	59.7%	70.8%	77.4%	80.7%	91.7%	97.4%
38	46.3%	52.4%	60.2%	71.2%	77.7%	81.0%	92.0%	97.5%
39	46.9%	52.8%	60.6%	71.7%	78.2%	81.3%	92.1%	97.6%
40	47.5%	53.4%	61.1%	71.8%	78.5%	82.2%	92.3%	97.6%
41	48.0%	53.8%	61.6%	72.4%	78.8%	82.5%	92.5%	97.7%
42	48.6%	54.2%	62.0%	72.8%	79.0%	82.7%	92.6%	97.7%
43	49.1%	54.8%	62.4%	73.1%	79.4%	83.0%	92.8%	97.8%
44	49.6%	55.2%	62.8%	73.5%	79.6%	83.2%	92.9%	97.8%
45	50.1%	55.7%	63.2%	73.8%	80.0%	83.5%	93.0%	97.9%
46		56.0%	63.6%	74.1%	80.2%	83.7%	93.2%	97.9%
47		56.5%	64.0%	74.4%	80.5%	84.0%	93.3%	98.0%

48	56.8%	64.3%	74.7%	80.6%	84.2%	93.3%	98.0%
49	57.3%	64.7%	75.0%	81.0%	84.4%	93.6%	98.1%
50	57.7%	64.8%	75.3%	81.1%	84.6%	93.6%	98.1%
51	58.0%	65.1%	75.6%	81.4%	84.8%	93.6%	98.1%
52	58.4%	65.4%	75.8%	81.7%	85.0%	93.8%	98.1%
53	58.8%	65.7%	76.1%	81.8%	85.2%	93.9%	98.2%
54	59.0%	66.0%	76.3%	82.1%	85.4%	94.1%	98.2%
55	59.4%	66.3%	76.6%	82.2%	85.5%	94.1%	98.2%
60	60.9%	67.7%	77.7%	83.2%	86.4%	94.5%	98.4%
65	62.4%	69.0%	78.7%	84.0%	87.1%	94.9%	98.5%
66	62.7%	69.2%	78.9%	84.2%	87.2%	94.9%	98.5%
67	62.9%	69.4%	79.1%	84.3%	87.4%	95.0%	98.6%
70	63.6%	70.1%	79.6%	84.7%	87.8%	95.2%	98.6%
72	64.1%	70.5%	80.0%	84.9%	88.0%	95.3%	98.6%
75	64.7%	71.1%	80.4%	85.4%	88.3%	95.5%	98.7%
80	65.8%	72.0%	81.2%	86.0%	88.8%	95.7%	98.7%
85	66.8%	72.8%	81.8%	86.5%	89.3%	96.0%	98.8%
90	67.6%	73.6%	82.4%	87.0%	89.7%	96.2%	98.8%
95	68.5%	74.3%	83.0%	87.4%	90.1%	96.3%	98.9%
100	69.2%	74.9%	83.5%	87.9%	90.5%	96.5%	99.0%
105	70.4%	75.6%	84.0%	88.2%	91.0%	96.6%	99.0%
110	70.6%	76.1%	84.4%	88.6%	91.1%	96.8%	99.1%
115	71.2%	76.7%	84.8%	88.9%	91.4%	96.9%	99.1%
120	72.4%	77.2%	85.2%	89.3%	91.7%	97.1%	99.2%
125	72.7%	77.6%	85.6%	89.5%	91.9%	97.1%	99.2%
130	72.8%	78.1%	85.9%	89.8%	92.1%	97.2%	99.3%
135	73.3%	78.5%	86.3%	90.0%	92.3%	97.3%	99.3%
140	73.8%	78.9%	86.6%	90.3%	92.5%	97.4%	99.3%
143	74.0%	79.1%	86.8%	90.4%	92.7%	97.5%	99.3%
144	74.2%	79.2%	86.8%	90.5%	92.7%	97.5%	99.3%
150	74.7%	79.6%	87.1%	90.7%	92.9%	97.6%	99.4%
155	75.4%	80.0%	87.4%	90.9%	93.0%	97.7%	99.4%
160	75.4%	80.3%	87.6%	91.1%	93.2%	97.8%	99.4%
165	75.7%	80.6%	87.9%	91.3%	93.4%	97.7%	99.4%
170	76.2%	80.9%	88.1%	91.5%	93.5%	97.8%	99.4%
175	76.5%	81.2%	88.3%	91.6%	93.6%	97.9%	99.4%
200	78.0%	82.5%	89.3%	92.4%	94.3%	98.2%	99.5%
210	78.5%	82.9%	89.6%	92.6%	94.5%	98.2%	99.5%
220	79.0%	83.3%	89.9%	92.9%	94.7%	98.3%	99.6%
225	79.2%	83.5%	90.1%	92.9%	94.7%	98.3%	99.6%
230	79.5%	83.7%	90.2%	93.1%	94.9%	98.4%	99.7%
240	79.9%	84.1%	90.5%	93.3%	95.0%	98.4%	99.7%
250	80.3%	84.4%	90.7%	93.4%	95.2%	98.5%	99.4%
260	80.7%	84.8%	90.9%	93.6%	95.4%	98.6%	99.5%
300	82.0%	86.0%	91.8%	94.2%	95.8%	98.7%	99.6%
350	83.4%	86.9%	92.6%	94.8%	96.3%	98.8%	99.7%
365	84.0%	87.3%	92.8%	95.0%	96.6%	98.9%	99.7%
400	84.5%	87.8%	93.2%	95.3%	96.7%	99.0%	99.8%
450	85.4%	88.6%	93.7%	95.7%	97.0%	99.1%	99.8%
500	86.2%	89.2%	94.2%	95.8%	97.2%	99.2%	99.8%
1000	90.4%	92.6%	96.5%	97.5%	98.4%	99.4%	99.9%
1100	90.9%	93.1%	96.7%	97.7%	98.5%	99.6%	99.9%

For a spreadsheet version of this table contact Dr Rodney Jones at hcaf_rod@yahoo.co.uk

Figure S1. Correlation between Clinical Commissioning Group avoidable ASMR versus matching all-cause ASMR for local authorities.

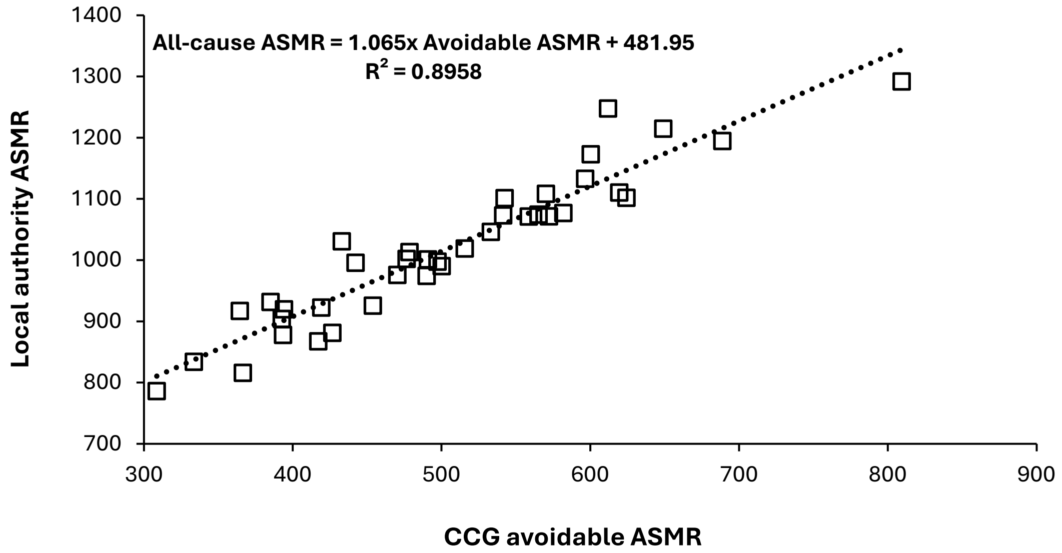

Figure S1. CCG avoidable ASMR versus Local Authority all-cause ASMR. Data from [17,18]. ASMR uses the European Standard Population.

In this correlation, CCG avoidable ASMR was first extrapolated to 2019 by linear regression of the avoidable ASMR between 2011 and 2019. This was then matched against the local authority ASMR in 2019. Some 46 CCGs had a matching local authority ASMR. The correlation in Figure S.1. was then used to estimate CCG all-cause ASMR where no matching local authority was available.

Figure S2. Standard populations for the World, Europe, and Australia.

In this section, the standard populations for the World, Europe, and Australia are compared using 5-year age bands. As shown in Figure S2 the World population is heavily weighted to under age 30, that for Australia to ages 25 to 54, while that for Europe to ages above 55. The calculated ASMR will therefore be weighted by the crude mortality rate in each age band relative to the standard population. The World ASMR is therefore weighted toward mortality experienced in children and young adults.

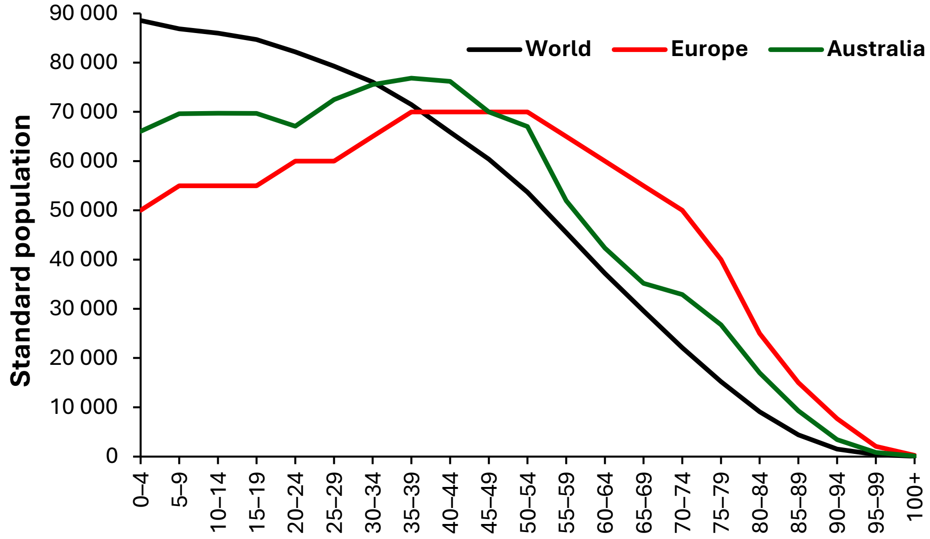

Figure S2. Standard populations for the World, Europe, and Australia. Data is from [55-56].
